# Letter from New York—Yellow Fever Prevented by Innoculation

**Published:** 1888-10

**Authors:** W. Miller

**Affiliations:** 149 East 12th Street, New York


### LETTER FROM NEW YORK—YELLOW FEVER PREVENTED BY INNOCULATION.

*To the Editor of the Southern Medical Record:*

SIR—In view of the grave consequences resulting from the scourge of yellow fever at Jacksonville, Fla., it seems fitting something ought to be done, more than has been done, to prevent the ravages of this contagion.

It is now generally understood by medical men, and those that have studied the ætiology of the disease, that it is a poison of the blood, and brought about by the inhalation of a poisonous disease germ, through the air currents, and also taken into the body, water, food, and possibly by absorption through the pores of the skin.

Whether these poisonous germs are the carriers of an infection, or fasten upon the internal organs of the body, and there multiply and reproduce themselves to an unlimited extent and then die and choke up the venous and arterial circulation, are questions yet to be determined. It has been shown that when this germ is taken into the organism by way of inhalation, they pass through the stomach with the food into the lower section of the intestinal canal without, harm where they reach the colon and find a suitable condition for their propagation, when within a few hours they multiply and reproduce themselves many millions.

This is the real beginning or incipient stage of yellow fever, and to clean out this obstruction and fermentive compact with epsom salts and castor oil, followed up with finely powdered fresh charcoal, is very effective treatment.

In contagious or infectious diseases the virulence depends upon the activity of the poison, and to modify this germ for the prevention or mitigation of disease, is now an interesting problem in medical science. That such a thing is possible, has been proven, or even shown that vaccination with a correctly prepared virus, will afford protection from so grave a malady as yellow fever. It would then seem incumbent on the medical profession to test the virtues of this prophylactic, without regard to explanations, whether it compares strictly with pathology, and save the lives of thousands who are or may be exposed to this malignant disease.

To prove the value of an antagonistic poison that is safe and harmless, it is now on record, of a medical professor who inoculated one thousand four hundred and thirty-eight persons exposed to yellow fever, *not one of whom died*, although seven were attacked with the disease. This is authentic, and other clinical evidence can be given in support of this theory and treatment of yellow fever by vaccination.

On July 28th, 1873, the first vaccination was performed in this country for hereditary tubercular consumption with success, and of later date for other diseases, by this mode of practice.

Since the discovery of the disease germ, and its relation to disease, enough has already been done to satisfy the public mind of the practicability of preventing yellow fever with a correctly modified virus, not so much for a cure, but a preventive, as small pox is prevented in man. Though this fact of preventing diseases has long been known to the scientific world, the difficulty has heretofore been as to the correct and safe method of attenuation. The day is not far distant when the application of distinct viruses for different diseases will be in general practice, and perhaps better understood for the relief of suffering humanity. Respectfully, W. MILLER,

New York, September, 1888. 149 East 12th Street, New York.

---

**Have** been using TONGALINE for two or three years with the most happy results and find it has excelled any other remedy for the cure of muscular rheumatism. J. M. JONES, M. D., Newport, Arkansas.

